# Clinical and economic evaluations of natalizumab, rituximab, and ocrelizumab for the management of relapsing-remitting multiple sclerosis in Saudi Arabia

**DOI:** 10.1186/s12913-023-09462-z

**Published:** 2023-05-26

**Authors:** Mansour A. Alharbi, Fahad Aldosari, Ahmed Hasan Althobaiti, Faris M. Abdullah, Salman Aljarallah, Nuha M. Alkhawajah, Miteb Alanazi, Yazed AlRuthia

**Affiliations:** 1 Department of Pharmacy, King Saud Medical City, Riyadh, 12746 Saudi Arabia; 2 Department of Neurology, King Saud Medical City, Riyadh, 12746 Saudi Arabia; 3 Department of Clinical Pharmacy, College of Pharmacy, Umm Al-Qura University, Makkah, 24382 Saudi Arabia; 4 Department of Medicine, Neurology Division, College of Medicine, King Saud University, P.O. Box 3145, Riyadh, 12372 Saudi Arabia; 5 Department of Pharmacy, King Khalid University Hospital, P.O. Box 3145, Riyadh, 12372 Saudi Arabia; 6 Department of Clinical Pharmacy, College of Pharmacy, King Saud University, P.O. Box 2454, Riyadh, 11451 Saudi Arabia; 7 Pharmacoeconomics Research Unit, Department of Clinical Pharmacy, College of Pharmacy, King Saud University, P.O. Box 2454, Riyadh, 11451 Saudi Arabia

**Keywords:** Multiple sclerosis, Relapsing-remitting, Natalizumab, Rituximab, Ocrelizumab, Cost effectiveness, Evidence based practice

## Abstract

**Introduction:**

The advent of new disease-modifying therapies (DMTs), such as monoclonal antibodies (mAbs), resulted in significant changes in the treatment guidelines for Multiple sclerosis (MS) and improvement in the clinical outcomes. However, mAbs, such as rituximab, natalizumab, and ocrelizumab, are expensive with variable effectiveness rates. Thus, the present study aimed to compare the direct medical cost and consequences (e.g., clinical relapse, disability progression, and new MRI lesions) between rituximab and natalizumab in managing relapsing-remitting multiple sclerosis (RRMS) in Saudi Arabia. Also, the study aimed to explore the cost and consequence of ocrelizumab in managing RRMS as a second-choice treatment.

**Methods:**

The electronic medical records (EMRs) of patients with RRMS were retrospectively reviewed to retrieve the patients’ baseline characteristics and disease progression from two tertiary care centers in Riyadh, Saudi Arabia. Biologic–naïve patients treated with rituximab or natalizumab or those switched to ocrelizumab and treated for at least six months were included in the study. The effectiveness rate was defined as no evidence of disease activity (NEDA-3) (i.e., absence of new T2 or T1 gadolinium (Gd) lesions as demonstrated by the Magnetic Resonance Imaging (MRI), disability progression, and clinical relapses), while the direct medical costs were estimated based on the utilization of healthcare resources. In addition, bootstrapping with 10,000 replications and inverse probability weighting based on propensity score were conducted.

**Results:**

Ninety–three patients met the inclusion criteria and were included in the analysis (natalizumab (n = 50), rituximab (n = 26), ocrelizumab (n = 17)). Most of the patients were otherwise healthy (81.72%), under 35 years of age (76.34%), females (61.29%), and on the same mAb for more than one year (83.87%). The mean effectiveness rates for natalizumab, rituximab, and ocrelizumab were 72.00%, 76.92%, and 58.83%, respectively. Natalizumab mean incremental cost compared to rituximab was $35,383 (95% CI: $25,401.09– $49,717.92), and its mean effectiveness rate was 4.92% lower than rituximab (95% CI: -30–27.5) with 59.41% confidence level that rituximab will be dominant.

**Conclusions:**

Rituximab seems to be more effective and is less costly than natalizumab in the management of RRMS. Ocrelizumab does not seem to slow the rates of disease progression among patients previously treated with natalizumab.

## Background

Multiple sclerosis (MS), is a complex neurodegenerative, inflammatory, progressive disease that results in multiple disabilities and is the most common disabling disease among young adults. [1, 2] Most patients show symptoms early in their 20s up to their late 30s, [2, 3] and females are more likely than males to experience MS symptoms. [4] According to the Global Burden of Disease (GBD) study, about 2.2 million people living with MS as of 2016, [5] and they are believed to reach 2.8 million in 2020. [4] The Middle East and North Africa (MENA) countries have low to moderate incidence rates of MS. However, recent studies indicate increasing incidence rates of MS in the MENA region. [6] This might be attributable to changes in people’s lifestyle in the MENA region and higher exposure to different environmental factors, such as the Epstein-Barr virus infection, obesity, smoking, and low vitamin D levels. [7] Among the member states of the Arabian Gulf Cooperation Council, the prevalence rate of MS is estimated to be more than 30 per 100,000 people, with Saudi Arabia having the highest prevalence of 40.40 per 100,000 people as of September 2018, according to a registry-based study that surveyed 20 hospitals in different regions of Saudi Arabia. [8] Relapsing-remitting MS (RRMS) is the most common type of MS, representing more than 80% of the diagnosed MS cases. [9] However, there are other types of MS, such as the primary– progressive MS (PPMS), secondary– progressive MS (SPMS), or progressive- relapsing MS (PRMS), which are not commonly seen in comparison to RRMS. Nevertheless, they have multiple signs/symptoms in common, such as fatigue, vision problems, mobility problems, and tingling or numb sensation. [10]

Due to the progressive and debilitating nature of MS, different medications have been approved to manage this disease and halt its progression. [11] However, none of the DMTs entirely prevents or reverse the progressive deterioration in the neurological functions of MS patients. [12] These medications are known as disease-modifying therapies (DMTs). They belong to different pharmacological classes, such as interferons (e.g., interferon beta-1b, interferon beta-1a), orally administered DMTs (e.g., fingolimod, siponimod, ozanimod, dimethyl fumarate, teriflunomide, and cladribine), and monoclonal antibodies (e.g., natalizumab, ocrelizumab, and alemtuzumab) which are commonly used in the management of MS in general, and RRMS in particular. [13] Monoclonal antibodies (mAbs) have proven their clinical superiority in comparison to other DMTs, such as interferons and orally administered therapies (e.g., fingolimod). [14–17] Rituximab and natalizumab are the most commonly utilized mAbs in the management of RRMS. They have demonstrated their effectiveness in reducing the rate of new lesion formation as demonstrated by Magnetic Resonance Imaging (MRI), physical disability, clinical relapse, and Expanded Disability Status Scale (EDSS) scores. [14, 16, 18–22] Although rituximab does not have a labeled indication for treating of RRMS, it has been widely used in different healthcare settings. [14, 20, 21] Moreover, it has shown comparable effectiveness and discontinuation rates to natalizumab. It has seemingly better long–term outcomes based on observational data from a single–center study in the United States (U.S.). [17] Furthermore, rituximab was associated with favorable relapse reduction compared to natalizumab based on a registry-based study in the U.S. that followed 204 MS patients on natalizumab and 115 patients on rituximab for 24 months. [23] In addition, rituximab has been shown to be more cost-effective than natalizumab in all of the simulations in a Markov–based model study that included 120 RRMS patients from Shiraz in Iran. [20] However, these studies were mainly based on observational data from a single center, limiting their results generalizability. On the other hand, ocrelizumab is a relatively new mAb that was approved by the USFDA in March 2017 and considered a better exit strategy for patients who failed natalizumab compared to other DMTs, such as fingolimod. [24] Moreover, it is considered a safe alternative therapy among natalizumab-treated patients at risk of progressive multifocal leukoencephalopathy (PML). [24–26] In a recently published study that evaluated different classes of DMTs for the management of RRMS in Saudi Arabia, mAbs (e.g., natalizumab and rituximab) were found to be the most effective DMTs in reducing the risk of physical disability progression, clinical relapse, and formation of new MRI lesions at 12 months follow–up compared to interferons (e.g., Rebif®), and orally administered DMTs (e.g., fingolimod, dimethyl fumarate, and teriflunomide). [27] However, no study has thus far examined the effectiveness and costs of individual mAbs in Saudi Arabia using real-world data. Therefore, this study aimed to compare the effectiveness of rituximab versus natalizumab in reducing the risk of disability progression, formation of new MRI lesions, and clinical relapse as well as their annual direct medical costs among RRMS patients. Moreover, the effectiveness and cost of ocrelizumab as a second-choice therapy for RRMS were evaluated.

## Methods

### Study design and population

This study was a retrospective review of electronic medical records (EMRs) that took place at two tertiary care centers (King Khalid University Hospital (KKUH) and King Saud Medical City (KSMC)) in Riyadh, Saudi Arabia. Ambulatory patients with relapsing-remitting multiple sclerosis (RRMS), and treated with mAbs (e.g., natalizumab, rituximab, ocrelizumab) for at least six months were recruited between May 2015 and February 2022. Patients with less than six months of follow–up, and those with other neurological disorders, such as epilepsy or Parkinson’s disease, or other types of MS (e.g., secondary progressive MS (SPMS) and primary progressive MS (PPMS)) were excluded. In addition, only biologic–naïve patients on natalizumab or rituximab were included, but not ocrelizumab since it is used as a second-choice therapy.

### Data collection

Sociodemographic characteristics (e.g., age and gender), medical characteristics (e.g., comorbidities), names, dosages, duration of therapy, and healthcare resource utilization (e.g., hospitalization, laboratory tests, imaging studies, emergency visits, etc.…) were retrieved from the EMRs of the neurology outpatient clinics in two tertiary care hospitals (KKUH and KSMC). Four pharmacists were involved in the data collection and three neurologists authenticated the retrieved data. No evidence of disease activity (NEDA-3) (i.e., absence of new T2 or T1 gadolinium (Gd) lesions as demonstrated by the Magnetic Resonance Imaging (MRI), disability progression, and clinical relapses) as adjudicated by the treating neurologists was used to assess effectiveness of mAbs. [28] The NEDA-3 was confirmed when no observable new T2 or T1 gadolinium (Gd) lesions were shown on the Magnetic Resonance Imaging (MRI), no return of old symptoms or worsening of current MS symptoms (i.e., clinical relapse), and no confirmed disability progression (CDP), such as increased walking difficulty leading to loss of independence as assessed by at least two neurologists (i.e., disability progression). The costs of different healthcare services (e.g., laboratory tests, imaging studies, medications, hospitalization, etc.…) were retrieved from the Saudi Ministry of Health cost center.

### Statistical analysis

Descriptive statistics using frequencies and percentages were used to present the baseline characteristics of the study sample. The individual rates of new lesions formation on MRI, disability progression, and clinical relapse, as well as the absence of disease progression (e.g., new T2 and/or T1 gadolinium (Gd) lesions on MRI, clinical relapse, and disability progression) were presented in percentages (%). In contrast, the mean annual direct medical costs (e.g., actual acquisition costs of prescription medications, hospital and emergency admissions, laboratory tests, and imaging studies, etc.…) for rituximab, natalizumab, and ocrelizumab were presented in United States Dollars ($). The direct medical costs and effectiveness rates of natalizumab and rituximab were compared using means and standard deviations. Additionally, inverse probability of treatment weighting using the propensity score based on patients’ characteristics, such as age, gender, duration of therapy, duration of illness, number of previous DMTs, and number of comorbidities was conducted. To address uncertainty with regard to cost and effectiveness difference between natalizumab and rituximab, non-parametric bootstrapping with 10,000 replications was conducted to generate the 95% confidence intervals (e.g., 95% CI). Ocrelizumab was not compared against rituximab or natalizumab since all patients on ocrelizumab were on natalizumab or rituximab and had treatment failure as demonstrated by new T2 and/or T1 gadolinium (Gd) lesions on MRI, clinical relapse, and disability progression. The minimum sample was estimated to be 70 patients based on the odds ratio (OR) of NEDA-3 of 2.5 for patients treated with natalizumab to their counterparts on rituximab, α = 0.05, β = 0.05, and power of 80%. [29] All statistical analyses were performed using SAS® version 9.4 (SAS® institute Inc, Cary, NC, USA).

## Results

**Fig. 1:**
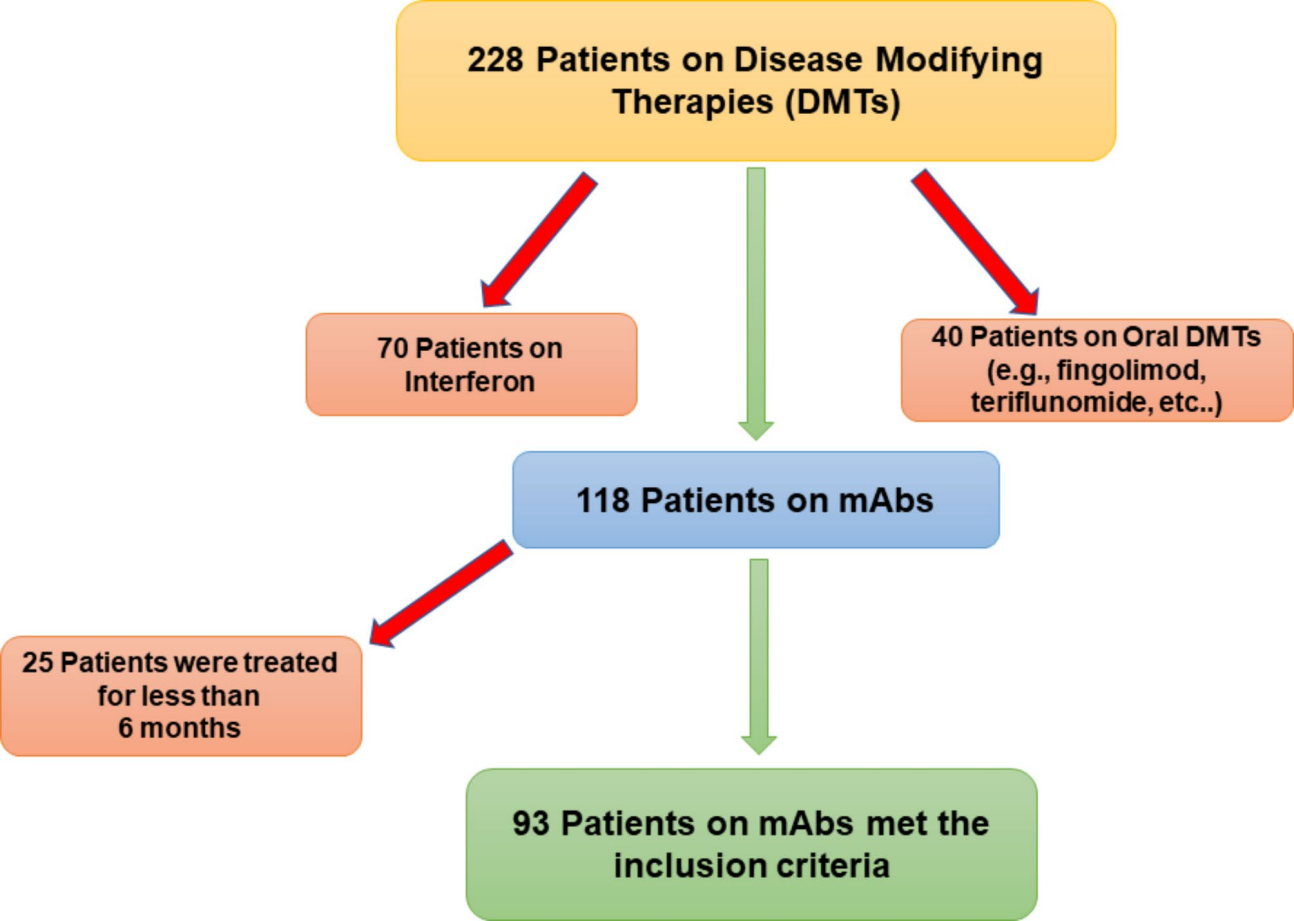
Flowchart of patients’ recruitment

**Fig. 2:**
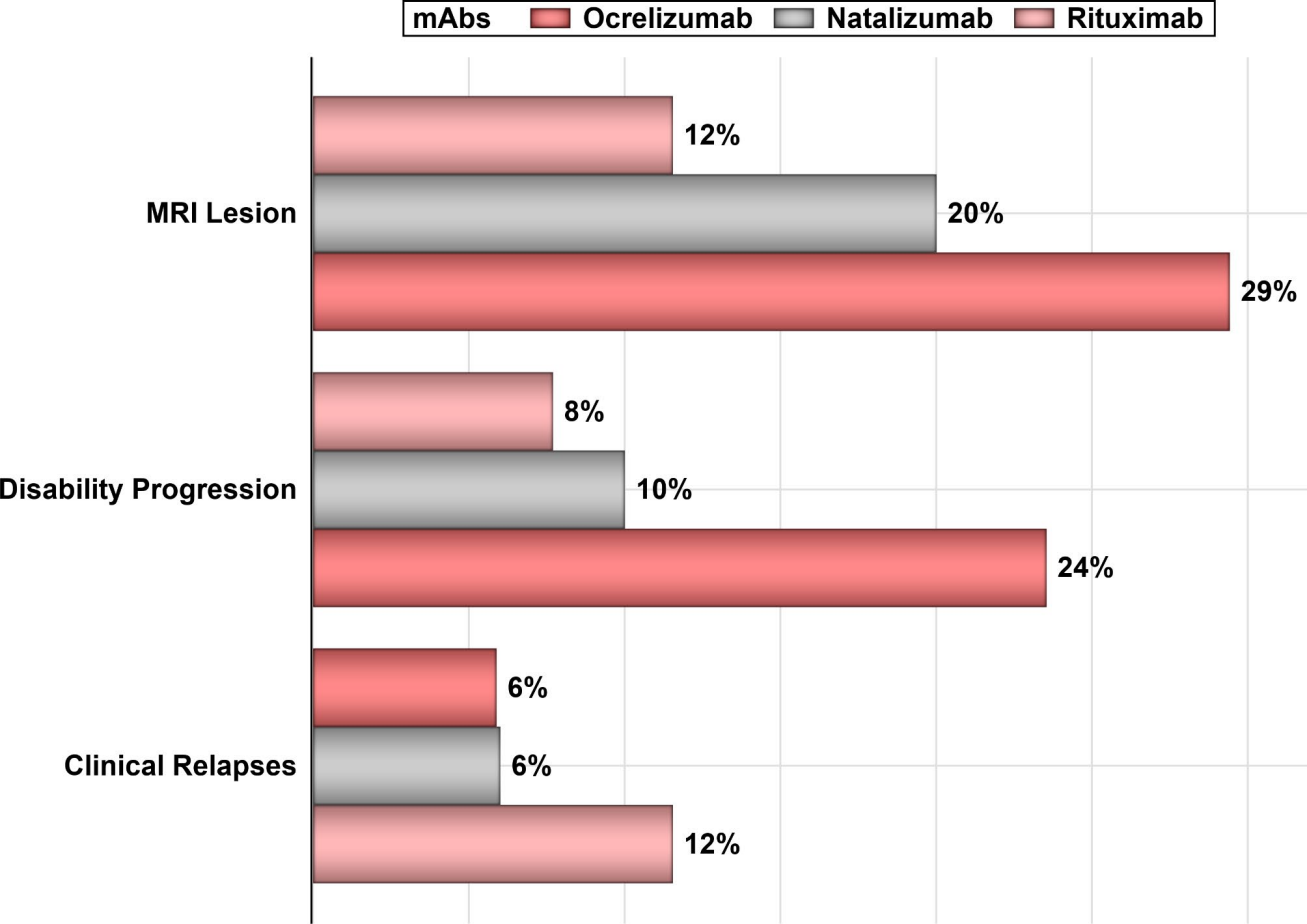
The mean rates (%) of disability progression, clinical relapse, and new lesions on MRI for patients on ocrelizumab, natalizumab, and rituximab

**Fig. 3:**
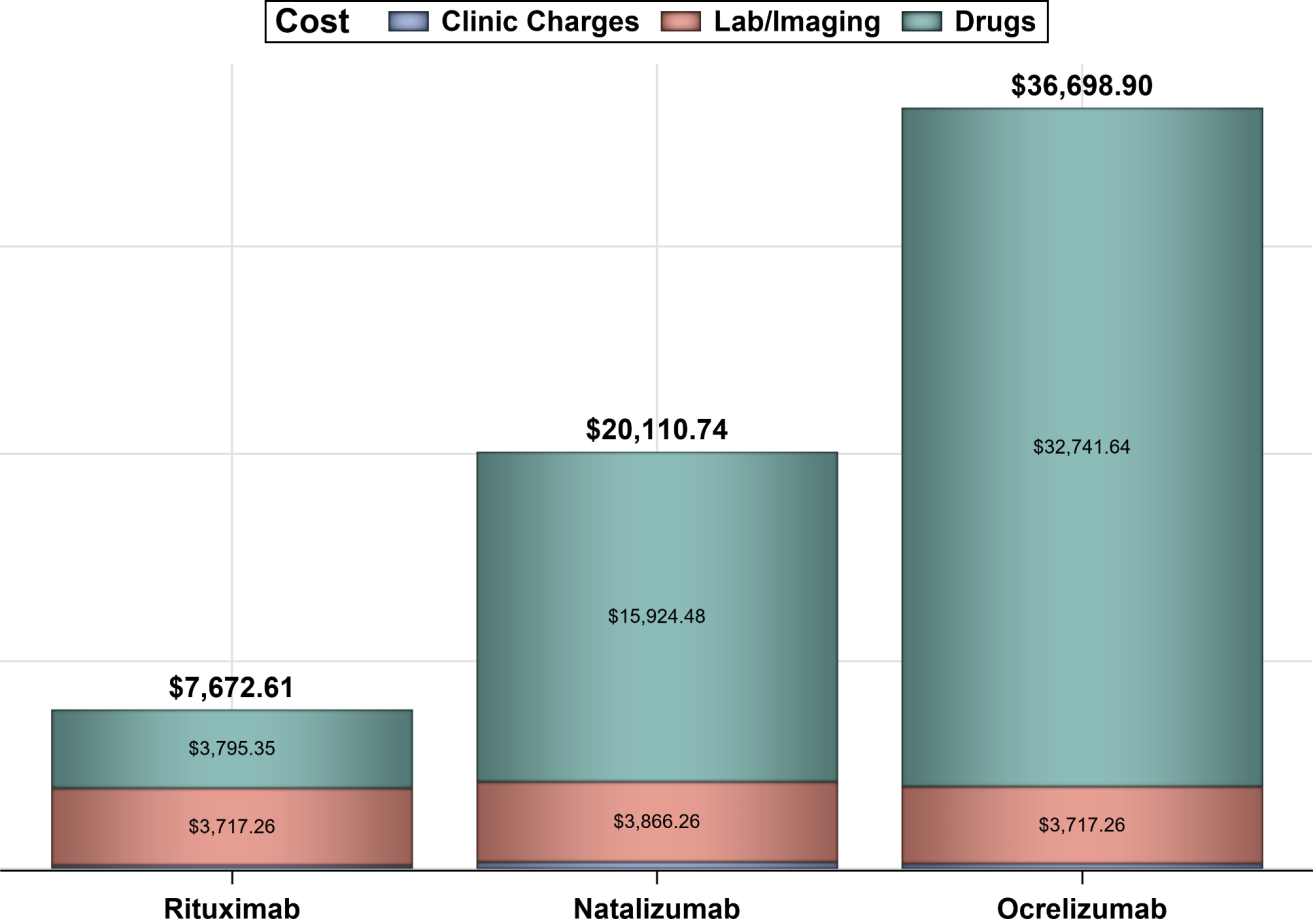
The mean annual costs of mAbs

Out of 228 patients’ EMRs that were reviewed, 93 patients met the inclusion criteria and were included in the analysis **(**Fig. 1**)**. About two–thirds of the patients were females (61.29%), and the majority were between 16 and 35 years of age (76.34%). Approximately 54% of patients were on natalizumab, 28% on rituximab, and 18% on ocrelizumab as shown in Table 1. Most of the patients were treated with mAbs for more than 1–year (83.87%), and only 18% of the patients had other health conditions, such as diabetes, hypertension, and dyslipidemia. The overall mean effectiveness rate as measured by NEDA-3 (i.e., absence of new T2 or T1 gadolinium (Gd) lesions as demonstrated by the Magnetic Resonance Imaging (MRI), disability progression, and clinical relapses) was 70.96% (95% CI: 61.56–80.36%); while the mean effectiveness rates for natalizumab, rituximab, and ocrelizumab were 72.00% (95% CI: 59.11 – 84.88%), 76.92% (95% CI: 59.57 – 94.27%), 58.83% (95% CI: 32.74 – 84.91%), respectively. The mean rates (%) of new lesions formation on MRI, clinical relapses, and disability progression for natalizumab, rituximab, and ocrelizumab are shown in Fig. 2. Although the rates of new lesions formation on MRI and disability progression among patients on natalizumab were higher in comparison to their counterparts on rituximab, this difference was not statistically significant (*p*–value = 0.4295). On the other hand, the rates of new lesions formation on MRI, disability progression, and clinical relapses for patients on ocrelizumab as a second-choice therapy were the highest in comparison to the biologic–naïve patients who were on either rituximab or natalizumab. The mean annual costs (e.g., drug acquisition cost, lab test, imaging studies, and clinic charges) for rituximab, natalizumab, and ocrelizumab were $7,672.61, $20,110.74, and $36,698.90, respectively, as shown in Fig. 3.

**Table 1:** Patient baseline characteristics (n = 93)

Characteristic	Rituximab (n = 26)	Natalizumab (n = 50)	Ocrelizumab (n = 17)	Frequency (%)
Gender, (n, %)				
Male	12(46.2)	16(32.0)	8(47.1)	36(38.7)
Female	14(53.9)	34(68.0)	9(52.9)	57(61.3)
Age, (n, %)				
16 yrs.—25 yrs.	15(57.7)	11(22.0)	1(5.9)	27(29.0)
26 yrs.—35 yrs.	7(26.9)	25(50.0)	12(70.6)	44(47.3)
36 yrs. —45 yrs.	3(11.5)	10(20.0)	2(11.8)	15(16.1)
> 45 yrs.	1(3.9)	4(8.0)	2(11.8)	7(7.5)
Duration of illness, (n, %)				
≤ 2 yrs.	13(50.0)	6(12.0)	0(0.0)	19(20.4)
2 yrs. – ≤4 yrs.	9(34.6)	8(16.0)	1(5.9)	18(19.4)
4 yrs. – ≤8 yrs.	4(15.4)	31(62.0)	11(64.7)	46(49.5)
> 8 yrs.	0(0.0)	5(10.0)	5(29.4)	10(10.8)
Duration of therapy, (n, %)				
6 months − 1 year.	5(19.2)	9(18.0)	16(94.1)	15(16.1)
1 year. – 2 yrs.	12(46.2)	14(28.0)	1(5.9)	42(45.2)
2 yrs. – 3 yrs.	5(19.2)	14(28.0)	0(0.0)	19(20.4)
> 3 yrs.	4(15.4)	13(26.0)	0(0.0)	17(18.3)
Number of used DMTs, (n, %)				
1	7(26.9)	49(98.0)	2(11.8)	58(62.4)
2	14(53.9)	1(2.0)	8(47.1)	23(24.7)
> 2	5(19.2)	0(0.0)	7(41.2)	12(12.9)
Other comorbid health conditions (e.g., hypertension, diabetes, dyslipidemia), (n, %)				
No	2(7.7)	42(16.0)	6(35.3)	16(17.2)
Yes	2(7.7)	8(16.0)	6(35.3)	16(17.2)

**Fig. 4:**
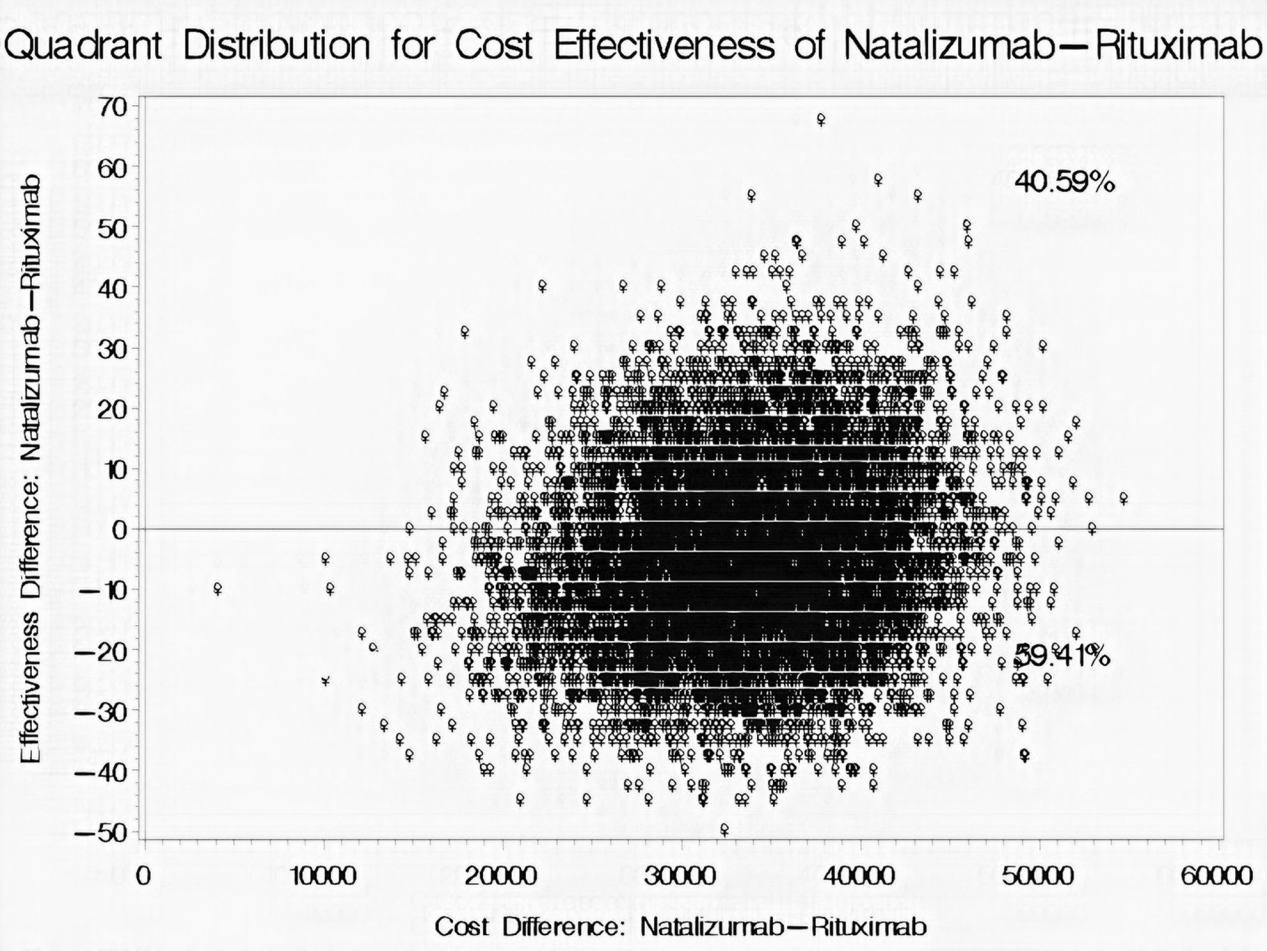
Bootstrap distribution of cost-effectiveness for natalizumab versus rituximab

The mean cost of treatment and effectiveness rates for both natalizumab and rituximab are shown in Table 2. Natalizumab was on average $35,383 (95% CI: $25,401.09– $49,717.92) more expensive with 4.92% lower effectiveness rate (95% CI: (-30–27.5) than rituximab. Additionally, the bootstrap distribution of cost effectiveness uncertainty shows that rituximab is more effective than natalizumab in 59.41% of the 10,000 replications, and less costly in all of the 10,000 replications adjusted for age, gender, number of comorbidities, duration of therapy, duration of illness, and number of previous DMTs as shown in Fig. 4.

**Table 2:** The mean effectiveness rates and costs of Natalizumab, and Rituximab

Difference in Cost and Effectiveness Rate between Natalizumab and Rituximab			
	Natalizumab	Rituximab	Mean difference (95% confidence interval)
Cost of treatment (USD), mean ± SD	50,843.5 ± 32,625.9	15,460.1 ± 12,969.2	35,383.4(25,401.1–49,717.9)
Effectiveness rate (%),^†^ mean ± SD	72.0 ± 45.4	76.9 ± 42.9	-4.92(-30–27.5)

^†^ Effectiveness is measured as the reduction in the rate of disease progression as assessed by NEDA-3. Costs included all direct medical costs (e.g., drug acquisition price, lab tests, imaging studies, etc.…) retrieved from the Saudi Ministry of Health cost center

## Discussion

The innovation in the MS treatment comes at a cost with great uncertainty about the incremental benefit of the new DMTs, such as the recently approved mAbs. [30, 31] Therefore, comparing the effectiveness rates and costs of different innovative DMTs, such as natalizumab, for the management of MS is essential to assess their incremental benefits vis-a-vis other cheaper alternatives, such as rituximab. [20] In this study, two commonly prescribed mAbs (e.g., natalizumab and rituximab) were compared concerning their effectiveness in preventing RRMS progression as defined by the formation of new lesions on MRI, clinical relapse, and disability progression using real-world data retrieved from two tertiary care centers in Saudi Arabia. Although natalizumab is costlier than rituximab and has a labeled indication for MS, this did not result in better effectiveness in most bootstrap cost effectiveness distributions. The incremental effectiveness rate of rituximab can be as high as 27.5% compared to rituximab with 59.41% confidence level that rituximab will result in better effectiveness rate adjusting for age, gender, duration of therapy, number of comorbidities, duration of illness, and number of previous DMTs. These findings are consistent with the results of a recently published study that compared rituximab to natalizumab in Iran using a Markov economic model with a lifetime horizon and found rituximab more cost-effective than natalizumab. However, their findings should be taken with a grain of salt due to the fact that the model was based on transition probabilities retrieved from different observational and experimental studies. Moreover, the Markov models have inherent limitations that make interpreting the results tricky. [32] In another study that included 740 RRMS patients treated with natalizumab or anti-CD20 drugs (e.g., rituximab or ocrelizumab) and were followed up for 24 months after fingolimod treatment failure in France, natalizumab was not found to be superior to rituximab or ocrelizumab concerning the clinical relapse, new lesions formation on MRI, or lower Expanded Disability Status Scale (EDSS) scores. [33] Moreover, the discontinuation rate after 18 months of treatment was higher among the patients on natalizumab compared to their counterparts on rituximab or ocrelizumab. [33] Additionally, real-world data on the use of rituximab for managing central nervous system’s demyelinating disorders in Italy has proven it safe. [21] Therefore, it might be wise to start patients on rituximab rather than natalizumab due to the uncertainty regarding the incremental benefit of natalizumab and only start natalizumab in case of rituximab failure. Another interesting finding in this study was the treatment failure rate among patients on ocrelizumab who were previously treated with natalizumab or rituximab. Approximately 40% of patients on ocrelizumab had a clinical relapse, disability progression, or new lesions on MRI. These findings shed light on the effectiveness of ocrelizumab compared to natalizumab and whether switching MS patients who failed natalizumab or rituximab to ocrelizumab is the right treatment strategy due to its higher acquisition cost. This finding is unsurprising since other studies which evaluated ocrelizumab as an exit strategy for natalizumab among patients with RRMS who are at high risk of PML found improved safety but similar effectiveness of ocrelizumab when compared to natalizumab. [34, 35] However, in a multicenter, retrospective, real-world data study that examined the effectiveness and safety of ocrelizumab versus rituximab among patients with RRMS in Italy who were previously treated with natalizumab, no difference was found in the rates of annualized relapse or safety profiles between those on ocrelizumab and their counterparts on rituximab. [35]

This study is the first, to the best of our knowledge, to examine the cost and effectiveness of commonly prescribed mAbs for managing of RRMS in Saudi Arabia using real-world data. However, some limitations of this study must be acknowledged. First, the effectiveness was not measured using the EDSS, the most widely used measure to assess the progression of MS. [36] This was mainly because very few practicing neurologists document the progression of their MS patients in the EMRs using the EDSS. Moreover, NEDA-3 (i.e., absence of new T2 or T1 gadolinium (Gd) lesions as demonstrated by the Magnetic Resonance Imaging (MRI), disability progression, and clinical relapses) was used instead of the health-related quality of life which is the commonly used measure in most cost effectiveness analyses. [37] Nonetheless, examining the effectiveness using clinical indicators, such as formation of new lesions on MRI or clinical relapse, is common and informing to the clinicians and policymakers. [27, 33] In addition, there is a link between the formation of new lesions on MRI, clinical relapse, and disability progression, as demonstrated in multiple clinical trials of MS patients. [38] The study included data from two tertiary care centers in Saudi Arabia, but the results are not generalizable to other healthcare settings. Furthermore, the study used fixed costs for different healthcare services, including prescription medications since the study was conducted from the perspective of public healthcare institutions that represents more than 60% of the healthcare coverage in Saudi Arabia. [39] In addition, no sensitivity analyses were conducted to check whether varying the costs of prescription drugs or other healthcare services, such as the MRI, would significantly change the results. Nevertheless, the findings of this study used very conservative and actual cost estimates of different healthcare services that were included in the model. Additionally, information bias cannot be ruled out since the study used data retrieved from the EMRs of two tertiary care hospitals. Moreover, no direct comparisons between rituximab or natalizumab and ocrelizumab were made since ocrelizumab is used as a second-choice therapy in these hospitals due to its higher cost. Furthermore, the rates of adverse drug events were not compared since they were not documented in the EMRs.

## Conclusions

Although the use of rituximab in the management of RRMS is off–label, it has been widely used and has shown a comparable effectiveness rate to natalizumab. However, natalizumab is considerably more expensive than rituximab with great uncertainty about its clinical superiority to rituximab. Moreover, the role of ocrelizumab as a second-choice treatment to natalizumab or rituximab should be examined further. Finally, future studies with larger sample sizes and more robust research designs should be conducted to validate the findings of this study.

## Data Availability

The datasets generated and/or analyzed during the current study are not publicly available due to the institutional review boards of King Khalid University Hospital and King Saud Medical City requirement of not publishing the raw data to the public. However, the de-identified raw data are available from the corresponding author (Yazed AlRuthia) on reasonable request.

## List of abbreviations

Ms: Multiple sclerosis
RRMS: Relapsing Remitting Multiple Sclerosis
DMTs: Disease Modifying therapies
mAbs: Monoclonal Antibodies
NEDA: No Evidence of Disease Activity
HRQoL: Health–Related Quality of Life
MRI: Magnetic Resonance Imaging
EMRs: Electronic Medical Records
MENA: Middle East and North Africa
PPMS: Primary Progressive Multiple Sclerosis
SPMS: Primary Progressive Multiple Sclerosis
PML: progressive multifocal leukoencephalopathy.
KKUH: King Khalid University Hospital
KSMC: King Saud Medical City
ICER: Incremental Cost Effectiveness Ratio
